# Large Structural Shear Deformation and Failure Monitoring Using Bend Losses in Polymer Optical Fibre

**DOI:** 10.3390/s20010195

**Published:** 2019-12-29

**Authors:** Terry Y. P. Yuen, Cheng-An Tsai, Trissa Deb, Yu-Hsiang Lin, June Nyienyi, Kai Tai Wan, Qunxian Huang

**Affiliations:** 1 Department of Civil Engineering, National Chiao Tung University, Hsinchu 30010, Taiwan; thesaviour09@yahoo.com.tw (C.-A.T.); trishadeb23@gmail.com (T.D.); st9001322@gmail.com (Y.-H.L.); junenyienyi@gmail.com (J.N.); 2 Sol-Bright LTD., Shatin, Hong Kong, China; kaitai.wan@sol-bright.com; 3 College of Civil Engineering, Huaqiao University, Xiamen 361021, China; huangqx@hqu.edu.cn

**Keywords:** distributed OFS, bend loss, large shear deformation, failure monitoring, POF, ν-OTDR

## Abstract

Rapid identification of structural damage positions is essential to the post-disaster rehabilitation of structures and infrastructures. Large shear deformation, e.g., shear failure of bridge piers, shear-slip of slopes, and shear cracking of structural walls, is often the cause of structural instability. Distributed optical fibre sensing (DOFS) techniques have an advantage over point-based sensors in terms of spatial continuous structural condition monitoring. This paper presents the development of new measurement theory and algorithm to evaluate the structural shear deflection based on the large beam deflection and optical bend loss theories. The proposed technique adopted a photon-counting Optical Time Domain Reflectometer (ν-OTDR) with polymer optical fibres (POFs) which has a large deformation measurement range and high spatial resolution. In the experiment, shear deformation events can be successfully detected and evaluated from the proposed technique. When the normalised shear deformation is larger than 0.2, both the event locations and the magnitudes can be accurately determined. When normalised shear deformation is lesser than 0.2, the error in the magnitude evaluation increased, but the event location can be found with an absolute error <0.5 m. Multiple shear events can be treated as independent events when they are separated by more than 5 m. Various configurations of POFs attached to concrete beam specimens for rupture failure monitoring were also studied. The configuration that could maximise the POF curvature at the beam failure produced the largest ν-OTDR signals. In other configurations in which the POFs were only stretched at failure, the signals were less strong and were influenced by the POF-structure bonding strength.

## 1. Introduction

Massive post-disaster rehabilitation and reconstruction program is a heavy burden to the governments of developing countries. The estimated cost of the post-2015 Nepal earthquake reconstruction program was equivalent to 25% of the country’s Gross Domestic Product (GDP) [1], and hundreds of thousands of earthquake victims were traumatized by the earthquake devastation. Furthermore, it may amount to a humanitarian crisis if the earthquake victims are not provided with sufficient temporary shelters as what tragically happened in the 2010 Haiti earthquake [2]. The relief of the disaster-affected areas would strongly depend on the rapidness and effectiveness of the rehabilitation of the damaged buildings. The sooner the severity of the structural damage can be assessed, the sooner the repair and reconstruction.

Sensors play a crucial role in detecting the health of the structure and accessing the probable damage or collapse. Over the past few decades, studies on optical fibre have started, consequently leading to the proposal of various ideas and techniques for structural health monitoring and damage detection [3,4,5,6,7,8,9,10,11]. Innovative distributed optical fibre sensors (DOFS) [9,12] are used over classical strain gauges to monitor the structural damage and preferred as a better alternatives because of the following reasons: (a) strain gauges are point sensors whereas DOFSs are continuous sensors along the entire length; (b) deployment of strain gauges requires the prior knowledge of expected damage locations; (c) installation of miniature strain gauges involves cumbersome gluing and wire welding processes; and (d) each strain gauge is connected to a pair of cables, and it can be quite disordered if a large number of strain gauges are required.

Habel and Bismarck [5] recommended the use of highly resolvable bare and plasma-polymer coated fibre sensors in concrete environment. These sensors could reliably measure the micro-deformation behaviour of special grouts. Abbiati et al. [6] proposed a prototype of an optical fibre sensor for monitoring civil engineering structures particularly steel structures to detect local bending of monitored structures. Song et al. [13] proposed an integrated distributed fibre optic sensing technology which includes Raman optical time-domain reflectometry, Brillouin optical time-domain analysis, and fibre Bragg grating sensing technology to monitor the temperature and variation of stress/strain of a pound lock reinforced concrete structure during the construction process. Wong et al. [9] successfully utilized distributed optical fibre sensors to monitor the damage growth in a pipeline subjected to transient hydraulic pressures.

The optical fibre sensing (OFS) techniques appeared to be maturing, and a number of field applications were implemented successfully [14]. Nonetheless, the above-mentioned OFS techniques were developed for single-mode fibres (SMF), of which most available in the market are predominantly made of glass or silicate. The fatal drawback of a silicate-based SMF is that it is brittle and prone to rupture under stress. The optical pulses cannot transmit through a breaking point of the SMF. The breaking strain of silicate-based SMF is typically around 3% to 4% only [15], and the typical elastic strain limit is 1% to 3% [16]. The SMF segment after the breaking point will lose its sensing ability entirely.

An alternative to silicate-based SMFs is polymer optical fibres (POFs), which are ductile and flexible under stress. Typical POFs can sustain the strain of more than 40% before rupture [17], which is unmatched by silicate-based SMFs. However, when the strain level exceeds 2%, the noticeable viscoelastic property of polymer has an influence on the strain measurement based on the Bragg wavelength shift [18]. Due to the viscoelastic effect, the strain-wavelength shift relationship would be no longer linear [18]. Another study [19] showed that if the strain of up to 3% is maintained for a few minutes before release, then the time-dependent viscoelastic effects would largely vanish and the reversible elastic deformation of the POF could be observed. Plastic deformation occurs in POFs after an approximate axial strain of 5% [16] that leads to the memory effect observed in the OTDR. This effect will not disappear even after the POF is unstrained [20], and it could be possibly used to measure the maximum stain under cyclic loading and long-term loading. The viscoelastic and plastic deformations may cause hysteresis effects and nonlinearity in the measurement but do not render POFs completely unusable in sensing provided that some compensation techniques [21] and calibrations are applied.

The experimental sensing characteristics of POFs under axial strain, bending, and twisting has been explored [20,22]. POFs can sustain large and cyclic deformation without significant changes in the material properties. By aligning the fibre to an appropriate angle with the crack plane, POFs can be used to monitor crack development in concrete structures [23]. Therefore, POFs would be particularly suitable for monitoring structures and infrastructures that may suffer extensive and multiple structural damages as in the case of earthquakes. Furthermore, large shear damage/rupture is well-known to be the major cause of the instability of buildings, bridge piers, and slopes [24,25], but shear deformation sensing with POFs has been scarcely studied.

In this regard, this paper presents the development of an optical sensing technique and theory using POFs and photon-counting Optical Time Domain Reflectometer (ν-OTDR) for large shear deformation and failure monitoring. The large beam deflection theory and the optical bend loss theory have been successfully combined to correlate the shear deflection magnitudes to the optical energy loss measured by the ν-OTDR. The feasibly of the proposed POF sensing technique on monitoring multiple large deflection events was also verified by tests. Lastly, various configurations of POFs attached to concrete beams for failure monitoring were also studied.

## 2. Theoretical Background of POF Sensing with ν-OTDR

### 2.1. Measurement Principles and Events

**Figure 1:**
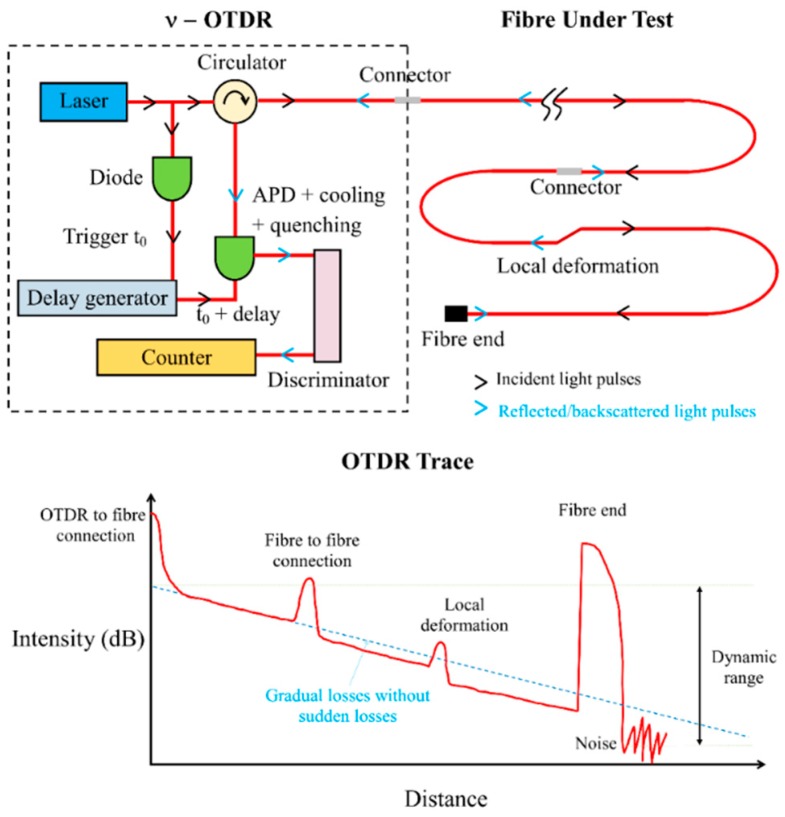
Working principles of photon-counting Optical Time Domain Reflectometer (ν-OTDR) [26] and typical OTDR trace.

The working principles of photon-counting Optical Time Domain Reflectometer (ν-OTDR) [26] are illustrated in Figure 1. The ν-OTDR sends out a train of light pulses (photons) with a fixed pulse width into the optical fibre. When encountering “events” (e.g., variation in the medium properties, boundaries, interfaces), the light pulses will be backscattered to the ν-OTDR that can measure the pulse intensity and locate the event distance based on the time intervals between the pulse generation and the pulse return. In this study, the light pulses have a wavelength of 650 nm and a pulse width of 1 ns travelling in a PMMA (Polymethyl–Methacrylate Resin) POF with a refractive index of 1.49026.

The sudden events in the OTDR traces (Figure 1) are caused by the local optical fibre deformation (e.g., strain or bending), connections (e.g., fibre-to-fibre or fibre-to-OTDR) or splices, fibre ends, etc. Except for splices, sudden events are reflective events, which cause reflection of the light pulses and a sharp increase in the light intensity in the OTDR trace at the location of the event. The peak of the reflected light intensity is related to the magnitude of the event. After the peak, the OTDR trace will decline gradually back to the gradual loss line but shifted down by the sudden loss (Figure 1). The characteristics of non-fusion spliced connector and deformation events are quite similar, but the reflection intensity of the former is often much higher due to the thin air layer at the interface. In practice, the locations of connectors are known beforehand and the portion of POFs used for sensing should be at least one meter away from the connections to avoid the dead zone as discussed below.

### 2.2. Modal Dispersion, Coupling, and Decoupling Effects on Event Measurement

The maximum spatial or sampling resolution of OTDRs for locating events in the optical fibre is dependent on the pulse width the smaller pulse width, the higher resolution, and the receiver recovery time [27]. However, a smaller pulse carries lower energy that challenges the detection power of the OTDR. Conventional OTDRs requires the photon flux with the minimum intensity of minus 70 dBm to make a measurement, but ν-OTDRs may only require minus 100 dBm photon flux [28]. The adopted ν-OTDRs in this study, of which specifications are provided in Table 1, can use a very small pulse width (e.g., 1 ns) to achieve high-resolution measurements.

Nevertheless, the pulse in an optical fibre can be broadened and distorted by the wavelength-dependent chromatic dispersion and the modal dispersion. In large-core multimode optical fibres, the modal dispersion effect dominates over the chromatic dispersion. The model coupling (energy is exchanged among different modes) is enhanced by random refractive-index perturbations which naturally present in POFs and that can reduce the modal dispersion [29].

The mechanical and optical properties of PMMA polymer can be affected significantly by physical aging, relative humidity, and temperature [30], in particular, when the temperature is larger than the glass transition temperature (*T_g_* = 108 °C for PMMA) [31]. The relative humidity can also influence the local attenuation and backscatter intensity changes [32]. Hence, the interaction of mechanical and relative humidity effects on the OTDR responses should be avoided if the sensor is designed to measure one physical property. Since the design service life of civil engineering structures is normally above 50 years, careful periodic recalibration of a POF monitoring system may be needed. Moreover, the high-temperature environment should be avoided and jacketed POFs can be adopted in a highly humid environment. Nevertheless, those features also make POFs a good temperature and humidity sensor. With the high thermal sensitivity (twice higher than silicate optical fibre (SOF) [33]), POF-Bragg gratings (POFBG) devices have been successfully applied for high-resolution temperature monitoring in biomedical areas [11].

**Figure 2:**
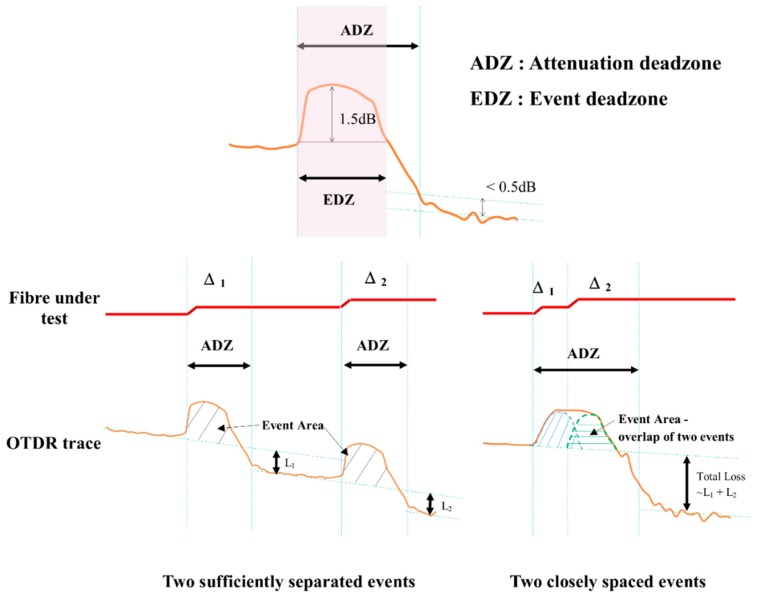
Dead zones and their effects on the measurement resolution.

The reflective events (e.g., connections and fibre deformation) can trigger the decoupling of higher-order modes (or mode filter effects) and event dead zones. The decoupling of higher-order modes can reduce the signal intensity of a successive event within the influencing length, which is dependent on the mode-coupling length in the POFs (~10 m for step-indexed POFs [35] and ~2 m for gradient-index POFs [29]). If a reflective event takes place within the event dead zone (EDZ) triggered by the preceding event, the location of the latter event cannot be pinpointed precisely. The width of the dead zone is influenced by the pulse width, the receiver recovery time, and the loss magnitude or return losses (RL) of the events. The event dead zone and its effects on the measurement by OTDR are illustrated in Figure 2.

### 2.3. Optical and Mechanical Properties of the Optical Fibre

The PMMA POFs with step refractive index profile adopted in this study consist of three parts: the PMMA core, the cladding, and the jacket. The core diameter is 1 mm where the light pulses travel. The cladding has a lower refractive index than the core. The assembly of the core and the cladding are protected by the jacket. Although the sensibility may be deceased, the jacket was not removed to prevent the scattering of ambient light into the fibre core through the cladding that can affect the ν-OTDR measurement. The properties of the PMMA POFs adopted in this study are summarised in Table 2.

## 3. Large Deformation Theory

### 3.1. Bend Loss in Large Shear Deformed Optical Fibre

**Figure 3:**
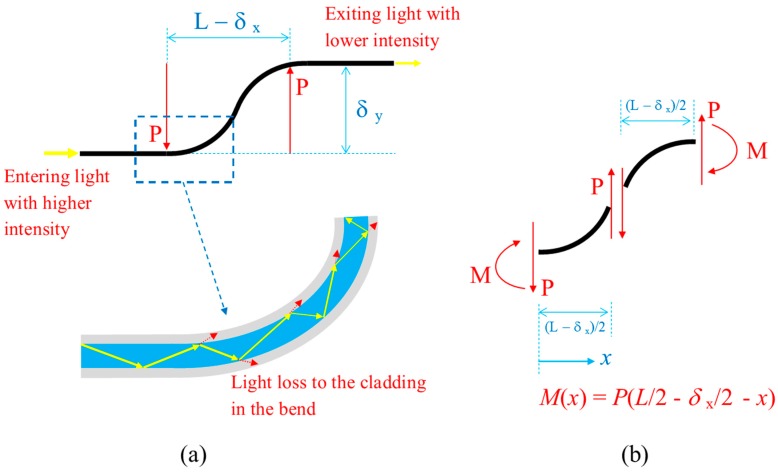
Shear deflection-induced bend loss in the optical fibre: (**a**) optical bend loss; (**b**) bending moment profile.

The responses of the POFs with the ν-OTDR to shear displacement are studied in this section. The energy loss or reflection of the light caused by the shear displacement is mainly due to the localised curvature known as the macro-bend loss that leads to the escape of light from the core to the cladding as illustrated in Figure 3. The bend loss for single-mode fibre can be computed by the classical Marcuse’s loss formula in terms of the superposition of cylindrical outgoing waves [36]. Marcuse’s loss formula was extended by Kaufman et al. [37] to compute the bend loss in the multimode optical fibres. However, a quite significant discrepancy can be observed in predicting the bend loss in multimode fibres using the analytical bend loss formulas. Hence, the semi-empirical relation for the bend loss adopted in this study which can be expressed as a function of the fibre’s curvature radius R:

(1) $$\alpha_{loss}=C_{1}\cdot R^{C_{2}}\cdot \exp\left(-C_{3}R\right)$$

where $C_{1}$, $C_{2}$, $C_{3}$ and are constants depending on the properties and geometries of the fibres which will be calibrated by the test data. Equation (1) is based on the formulation given in Senior and Jamro [37], but the original constant coefficient is modified to be dependent on R that better agrees with the experiment results. $\alpha_{loss}$ is the power loss coefficient:

(2) $$10\log_{10}\frac{P\left(0\right)}{P\left(x\right)}=\alpha_{loss}x$$

which describes the decay of the light power from P(0) to P(x) over a distance x. The power loss coefficient $\alpha_{loss}$ in Equation (2) is in units of decibel/unit-length.

To correlate the optical bend loss with the shear displacement, the curvature profile of the deformed POF segment shall be evaluated. The large deflection beam theory is employed here, and the curvature-bending moment relationship can be described by the classic Bernoulli–Euler equation:

(3) $$\frac{1}{R}=\frac{d\phi }{ds}=\frac{M\left(x\right)}{EI}$$

where ϕ is the tangential angle, s is the arc-length, M is the bending moment, and EI is the flexural stiffness of the beam which can be assumed constant throughout the optical fibre. The POFs is assumed to deform freely in the horizontal direction (i.e., horizontal load and axial strain are zero), then the bending moment follows a linear profile as:

(4) $$M\left(x\right)=P\left(L/2\;-\delta_{x}/2\;-x\;\right)$$

The end horizontal deflection $\delta_{x}$, the end vertical deflection $\delta_{y}$, and force P can be expressed in forms of the following integral equations [38].

(5) $$\alpha =\frac{1}{\sqrt{2}}\int_{0}^{\phi_{0}}\frac{d\phi }{\sqrt{\sin\phi_{0}-\sin\phi }};\;\frac{\delta_{y}}{L}=\frac{1}{\alpha \sqrt{2}}\int_{0}^{\phi_{0}}\frac{\sin\phi \;d\phi }{\sqrt{\sin\phi_{0}-\sin\phi }};\;\frac{\delta_{x}}{L}=\frac{1}{\alpha \sqrt{2}}\int_{0}^{\phi_{0}}\frac{\cos\;\phi \;d\phi }{\sqrt{\sin\phi_{0}-\sin\phi }}$$

where $\alpha =\sqrt{PL^{2}/\left(EI\right)}$ is the force index, $\phi_{0}$ is the angle of slope at the “cantilever free end” or the midpoint (x=L/2) of the whole deformed POF segment as illustrated in Figure 3. The above integral equations can be transformed into elliptic integrals [38] which can then be evaluated by infinite series:

(6) $$\alpha =F\left(\frac{\pi }{2},k\right)-F\left(\gamma ,k\right);\;\frac{\delta_{y}}{L}=1-\frac{2}{\alpha }\left[E\left(\frac{\pi }{2},k\right)-E\left(\gamma ,k\right)\right];\;\frac{\delta_{x}}{L}=1-\frac{\sqrt{2}}{\alpha }\sqrt{2k^{2}-1}.$$

in which F(γ,k) is an incomplete elliptic integral of the first kind with amplitude γ and modulus k and E(γ,k) is an incomplete integral of the second kind with amplitude γ and modulus k. The following transformations were used to convert Equation (5) to the above elliptic integrals:

(7) $$\sin\phi \;=2k^{2}\;\sin^{2}\theta \;-1;\;k=\sqrt{\left(1+\sin\phi_{0}\right)/2};\;\gamma =\sin^{-1}\sqrt{1/\left(1+\sin\phi_{0}\;\right)}.$$

In the above equations $\phi =\phi_{0}\;\mathrm{when}\;\theta =\pi /2$. It can be seen that the amplitude γ and modulus k needed to evaluate the elliptic integrals depend on $\phi_{0}$ only. The current problem setting is that the vertical end deflection $\delta_{y}$ was measured from the tests, and hence, Equations (6) and (7) can be used to determine $\phi_{0}$, the end horizontal deflection $\delta_{x}$ and the force P. Lastly, the moment M and curvature 1/R distributions using Equations (3) and (4) can be evaluated. The constants $C_{1}$ and $C_{2}$ in Equation (1) can be calibrated against the measured loss from the ν-OTDR traces. The effective bending stiffness EI of concentric tubes is

(8) $$EI=\overset{N}{\underset{j=1}{\sum }}\frac{\pi \left(r_{o,j}^{4}-r_{i,j}^{4}\right)}{4}E_{j}$$

where $r_{o,j}$ and $r_{i,j}$ are the outer and inner radii, respectively, of the *j*-th layer. $E_{j}$ is Young’s modulus of the *j*-th layer. The Young’s modulus E of each layer of the POFs is provided in Table 2 and the bending stiffness computed by Equation (8) is 1.0231 kN·mm^2^.

### 3.2. OTDR Trace Characteristics of Single Shear Events

**Figure 4:**
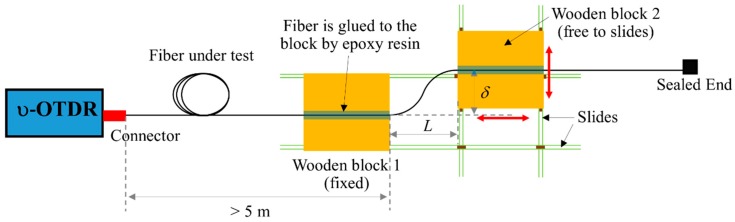
Shear test set-up.

The loss in the POFs due to a large shear deflection at a single position is first studied. Five different gauge lengths: 10 mm (S10), 15 mm (S15), 20 mm (S20), 40 mm (S40), and 80 mm (S80) are considered here. The shear test set-up is shown in Figure 4. The nomenclature of the tests is illustrated as follows: S10-10 is a test with a gauge length of 10 mm under a relative shear deformation of 10 mm.

To reduce the signal noise, the smoothing spline ${\bar{I}}_{i}\left(x_{i}\right)$ of the measured intensity $I_{i}\left(x_{i}\right)$ is first constructed by minimising the following function:

(9) $$\min\left[p\underset{i}{\sum }{\left(I_{i}-{\bar{I}}_{i}\right)}^{2}+\left(1-p\right)\int {\left(\frac{d^{2}{\bar{I}}_{i}}{dx^{2}}\right)}^{2}dx\right]$$

The smoothing parameter p has a value between 0 and 1. In this study, p=0.9999 is applied to obtain a sufficiently smooth spline fit. Before encountering the shear deformation, the traces follow the same straight attenuation curve. Then, the shear displacement triggered a sudden event in the trace and the mode decoupling.

**Figure 5:**
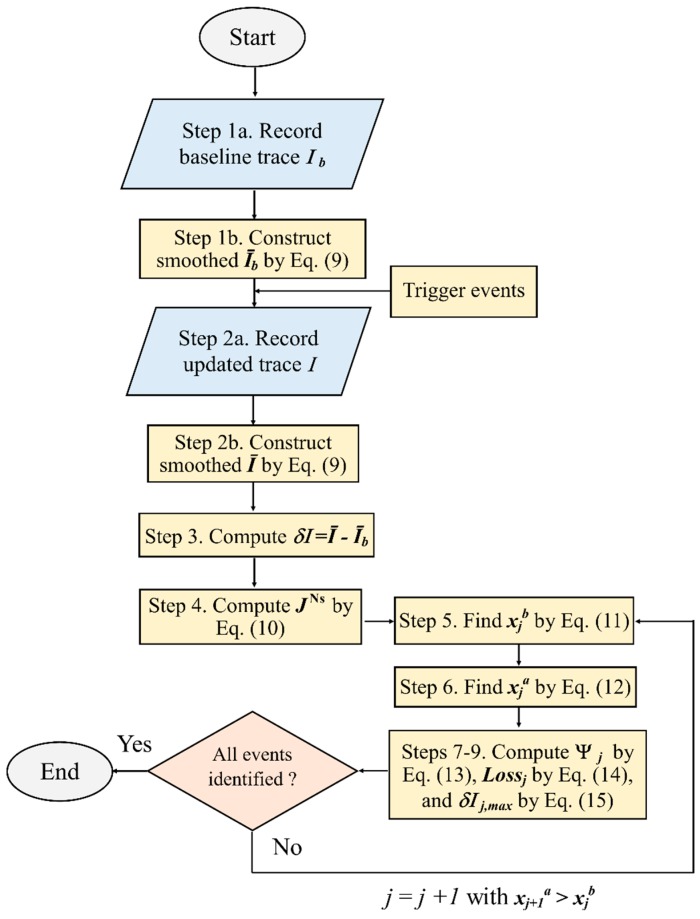
Flow chart for OTDR event characterisation process.

A robust method to define and characterise multiple events in an OTDR trace is proposed here as summarised in Figure 5 and described as followings:

Step 1: The initial OTDR trace $I_{i}^{b}\left(x_{i}\right)$ is recorded and then smoothed by Equation (9) to get the baseline trace ${\bar{I}}_{i}^{b}\left(x_{i}\right)$.

Step 2: After shear events are triggered, the updated $I_{i}\left(x_{i}\right)$ is recorded and smoothened to get ${\bar{I}}_{i}\left(x_{i}\right)$ by Equation (9).

Step 3: Compute the intensity change $\delta {\bar{I}}_{i}\left(x_{i}\right)={\bar{I}}_{i}\left(x_{i}\right)-{\bar{I}}_{i}^{b}\left(x_{i}\right)$.

Step 4: Compute the tangents of the intensity change $J_{i}^{N_{s}}\left(x_{i}\right)$ using linear regression with $N_{s}=2,3,\ldots ,8$ by

(10) $$\min\left[\overset{\alpha +N_{s}}{\underset{i=\alpha }{\sum }}{\left(J_{\alpha }^{N_{s}}x_{i}+d_{\alpha }^{N_{s}}-\delta {\bar{I}}_{i}\right)}^{2}\right]$$

Step 5: The endpoint $x_{j}^{b}$ of the j-th event can be defined as the points of the $J_{i}^{N_{s}}$ curves defined by Equation (10), for $N_{s}=1,2,\ldots m$, converging within a small error ϵ, i.e.,

(11) $$\sum_{N_{s},N_{s}^{\prime }=1}^{m}\left|J_{j}^{N_{s}}\left(x_{j}^{b}\right)-J_{j}^{N_{s}^{\prime }}\left(x_{j}^{b}\right)\right|<\epsilon \;x_{j}^{b}>x_{j}^{a}\text{ \& all }J_{j}^{N_{s}}\geq 0$$

Step 6: The beginning point $x_{j}^{a}$ of the j-th event is identified by the local maxima of $J_{j}^{N_{s}=3}$ as:

(12) $$\frac{\partial J_{j}^{N_{s}=3}\left(x_{j}^{a}\right)}{\partial x}=0\;\&\;\frac{\partial^{2}J_{j}^{N_{s}=3}\left(x_{j}^{a}\right)}{\partial x^{2}}>0\;x_{j}^{b}>x_{j}^{a}>x_{j-1}^{b}$$

Step 7: Compute the event area $\Psi_{j}$ of the *j*-th event zone defined as

(13) $$\Psi_{j}=\overset{x_{j}^{b}}{\underset{x_{j}^{a}}{\int }}\;\langle \delta \bar{I}\rangle \cdot dx$$

The Macaulay brackets $\langle \delta \bar{I}\rangle =\left\{\delta \bar{I},\delta \bar{I}>0;\;0,\;0\leq \delta \bar{I}\right\}$ are used to ensure Equation (13) is always positively defined.

Step 8: Compute the loss of the *j*-th event at $x_{j}^{b}$

(14) $$Loss_{j}=\delta \bar{I}\left(x_{j}^{b}\right)-\overset{j-1}{\underset{i=1}{\sum }}Loss_{i}$$

In Equation (14), the loss due to the preceding *j*−1 events is subtracted.

Step 9: The maximum intensity difference $\delta {\bar{I}}_{max}^{\prime }$ with the adjustment of the total previous loss due to reflection is computed by Equation (15), and the corresponding position $x_{j}^{c}$ is determined ($x_{j}^{b}>x_{j}^{c}>x_{j}^{a}$).

(15) $$\delta {\bar{I}}_{max}^{\prime }\left(x_{j}^{c}\right)=\delta {\bar{I}}_{max}\left(x_{j}^{c}\right)+\overset{j-1}{\underset{i=1}{\sum }}Loss_{i}$$

The normalized error Err is computed as

(16) $$Err=\frac{\left|x_{OTDR}-x_{L}\right|}{\sqrt{U_{OTDR}^{2}+U_{L}^{2}}}$$

where $U_{OTDR}$ and $U_{L}$ are the expanded uncertainties of the OTDR measurement and the physical length measurement, respectively. The signal-to-noise ratio (SNR) that depends on the OTDR device and the adopted POF would decrease over length due to the mode dispersion and affect the detection of small deformation events. Hence, the noises must be checked for long-distance measurement.

**Figure 6:**
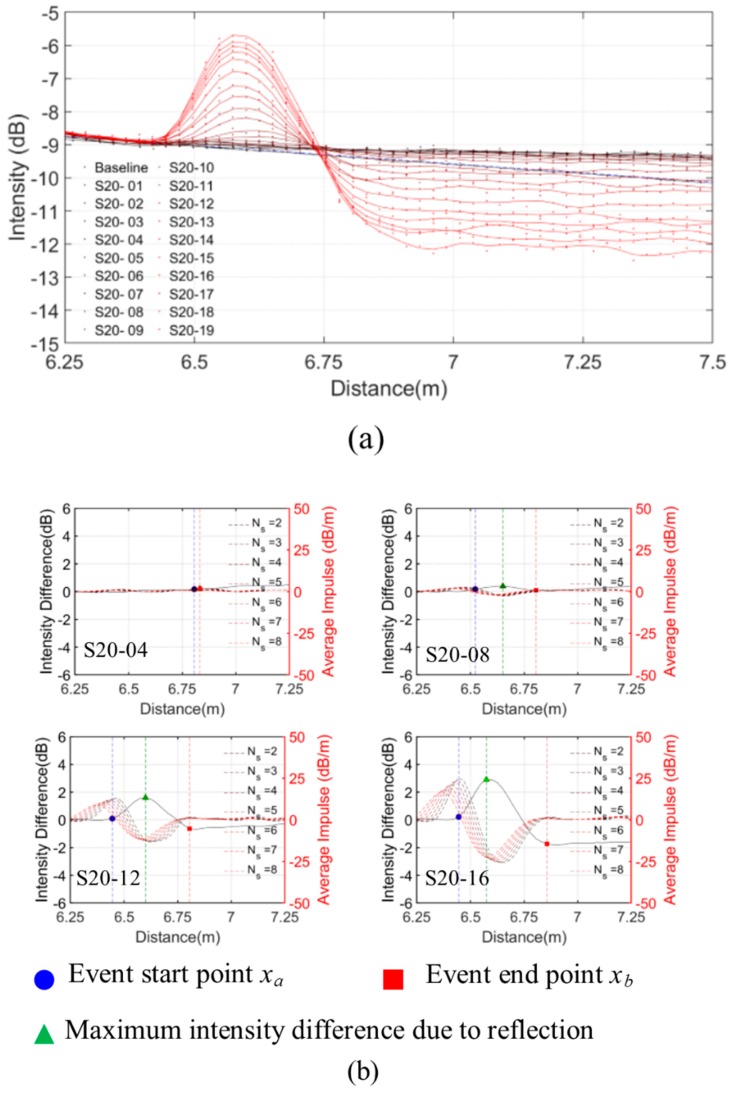
Single shear event with a deformed length of 20 mm: (**a**) ν-OTDR traces; (**b**) locating the event start points, the endpoints, and the maximum reflection intensity using the intensity difference (left axis) methods with the impulse for different $N_{s}$ (right axis).

As an example, the ν-OTDR traces for different magnitudes of normalised shear displacements for a deformed length of 20 mm are shown in Figure 6, which shows that the proposed technique can successfully and consistently characterise the events.

**Figure 7:**
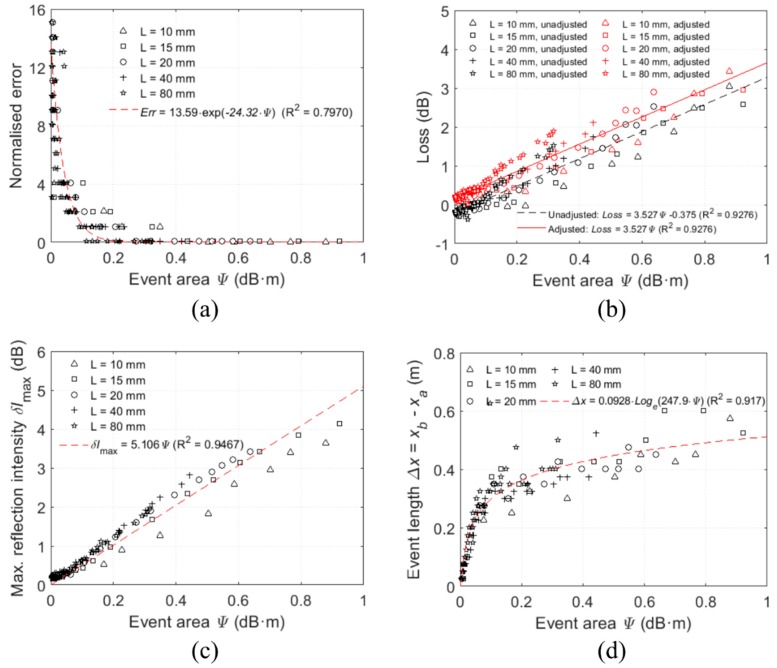
Correlations between event area and other variables: (**a**) Normalised error in detecting the shear event positions; (**b**) energy loss of the light pulses; (**c**) maximum intensity due to reflection; (**d**) event lengths due to shear deflections.

As shown in Figure 7b, the event area is linear with the loss and the non-zero intercept on the axis of the loss is the expected error due to the mismeasurement. The event area is also linear with the maximum linear intensity (Figure 7c). This can be adjusted by subtracting the originally measured loss by the intercept of the event area vs loss plot. The adjusted loss of each test is plotted in Figure 7b. Moreover, it can be observed from Figure 7a, the larger the event area produced, the more accurate in locating the deflection events. When the event area is larger than 0.2 dBm, the normalised error is generally less than 0.1. Despite the higher normalised error for small deflection events, the proposed technique can detect and locate the events with an error within 0.5 m which is generally acceptable for monitoring of large-scale civil engineering structures. To calibrate the constants $C_{1}$, $C_{2}$, and $C_{3}$ of the power loss coefficient given by Equation (1), the analytical total loss $Loss_{a}$ over the deformed length, L is evaluated by

(17) $$Loss_{a}=2\int_{0}^{L/2}\alpha_{loss}\cdot ds$$

The anti-asymmetry about the mid-span as shown in Figure 3 is used in the above equation. Using Equations (3) and (4), and dx/ds=cosϕ, the analytical total loss can be computed as

(18) $$Loss_{a}=2\int_{0}^{\frac{L-\delta_{x}}{2}}C_{1}\cdot {\left(EI/M\left(x\right)\right)}^{C_{2}}\cdot \exp\left(-C_{3}\cdot EI/M\left(x\right)\right)/\cos\phi \left(x\right)\cdot dx$$

In this study, the four-point Newton–Cotes quadrature rule is adopted to evaluate Equation (18).

**Figure 8:**
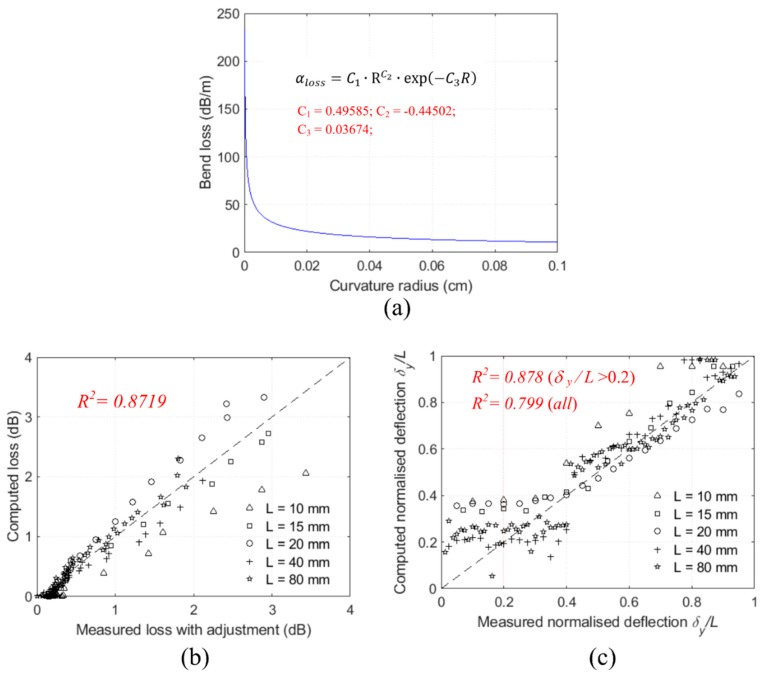
(**a**) The bend loss vs curvature radius relationship and the comparisons of the computed with the measured (**b**) loss and (**c**) normalised deflections.

The constants $C_{1}$, $C_{2}$, and $C_{3}$ of the power loss coefficient $\alpha_{loss}$ fitted against the measured losses with the adjustment by nonlinear least-square fitting are $C_{1}=0.49858$, $C_{2}=-0.44502$, and $C_{3}=0.03674$ (units: dB, mm, kN). The relationship between the power loss coefficient and the POF curvature is plotted in Figure 8a. The measured losses and the computed losses by Equation (18) are compared in Figure 8b.

For structural monitoring, the normalised deflection is to be estimated based on the measured loss and the gauge length which is the inverse problem of Equation (18). The measured normalised vertical deflections and the computed vertical deflections are compared in Figure 8c. The computed deflections are very close to the measured deflections when the deflections are sufficiently large, say $\delta_{y}/L>0.2$. As shown in Figure 6, even small deflections (intensity change > 0.1 dB) can be detected by the POF sensing system with reasonable estimations of the event positions. Nevertheless, the magnitudes of smaller deflection events $\delta_{y}/L<0.2$ cannot be accurately evaluated. Due to the mode dispersion, the signal-to-noise ratio is lower for long-distance measurements that further affect the detection and evaluation of small deflection events.

### 3.3. OTDR Trace Characteristics of Double Shear Events

**Figure 9:**
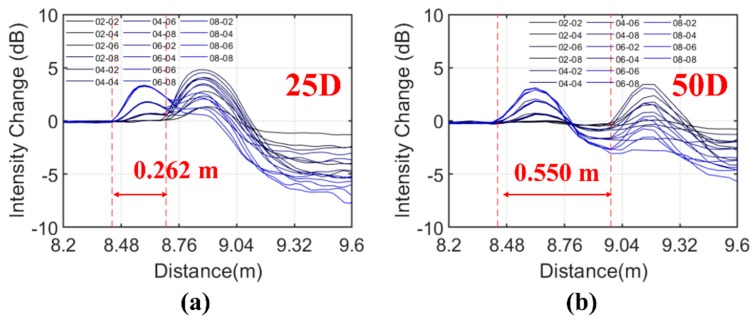

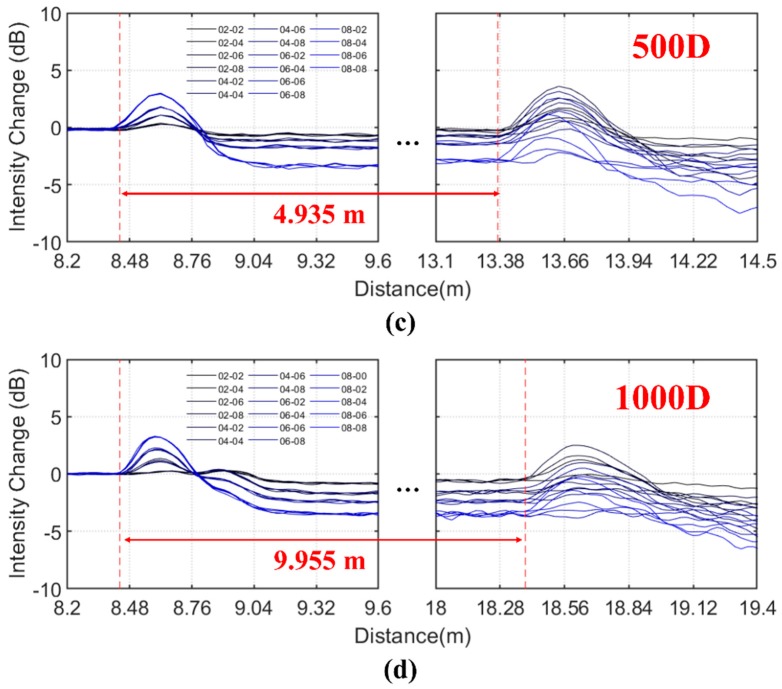
Intensity changes of double shear events with separation distances of (**a**) 25 cm, (**b**) 50 cm, (**c**) 500 cm, and (**d**) 1000 cm.

**Figure 10:**
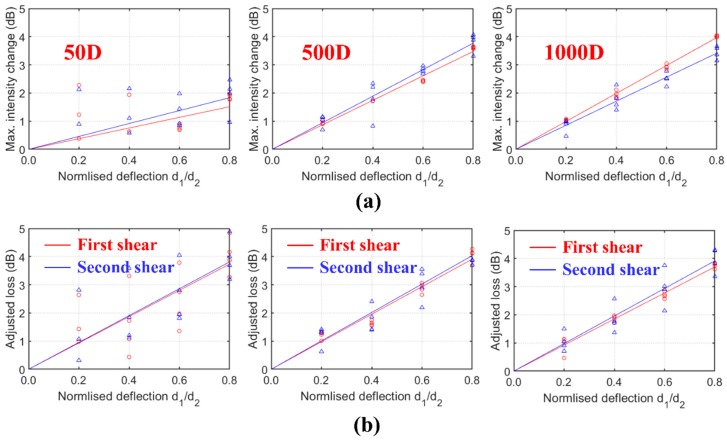
Correlations between shear deflections with (**a**) maximum intensity change due to reflection and (**b**) loss of individual shear events (∆= measured properties of the second shear events and ○= measured properties of the first shear events).

Closely spaced events will affect each other, and this increases the difficulty in characterising the properties of individual events from the OTDR signals. For continuous structural monitoring, the sensing technique must have the ability to detect multiple events. To this regard, the OTDR signals under double shear events with separations of 25 cm, 50 cm, 5 m, and 10 m denoted as test series 25D, 50D, 500D, and 1000D, respectively, are investigated in this section. The deformed length was 20 mm. The nomenclature of the tests is illustrated as follows: 25D-04-06 is a double shear test with a separation of 25 cm, the normalised deflection magnitudes of the first *d*_1_ and the second shear *d*_2_ are 0.4 and 0.6, respectively. The tested intensity changes in all test series are summarised in Figure 9. The relationships between the loss and maximum intensity changes with the individual deflection magnitudes are summarised in Figure 10.

The light modes decoupled and strongly backscattered at the deformed length. When the two events are too close, significant spatial superposition of closely spaced backscatter events of the light modes can occur. As shown in Figure 9a, the resulted OTDR signals of the two shear events in series 25D partly overlap each other. The second event could be hardly located by the proposed technique when the first shear magnitude was larger than the second shear magnitude (e.g., *d*_1_ > 0.6 and *d*_2_ < 0.4). The loss due to the first shear was cancelled by the reflection due to the second shear and, hence, only the total loss after the second shear can be evaluated. Although the positions of the two shear events can be clearly identified in test series 50D with separation of 0.5 m, the effects of decoupling and superposition of backscatter events that caused high variations in the deflection–loss and deflection–reflection intensity relationships of individual events were still quite significant (Figure 10).

When the separation increased above 5 m (test series 500D and 1000D), the mode decoupling effects reduce significantly. The two shear events can be treated as isolated events and analysed separately. The variations in the deflection–loss and deflection–reflection relationships (Figure 10) are lower compared to test series 50D.

**Figure 11:**
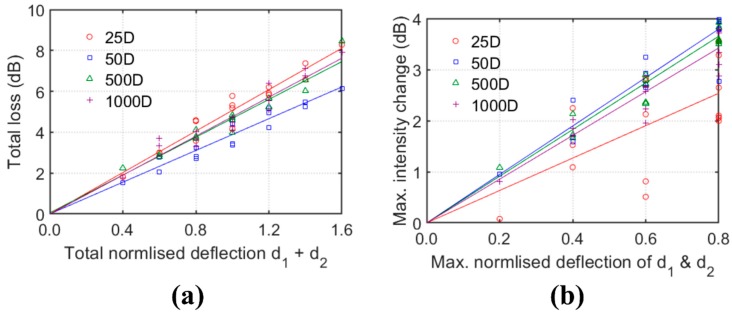
Relationships of (**a**) total loss vs. total normalised deflection and (**b**) maximum intensity change vs maximum normalised deflection.

Nevertheless, the total loss shows a very good and linear relationship with the sum of the two shear magnitudes (*d*_1_ + *d*_2_) as shown in Figure 11a. Even under significant effects of decoupling and superposition of backscatter events, the linear correlation is still very strong for 25D and 50D series. However, under a given total deflection magnitude, the total loss in the 25D or 50D series deviated from the total loss in the 500D or 1000D series (Figure 11a). Such deviation which increases with the total deflection magnitude could be caused by the mode decoupling.

The maximum intensity change due to reflection can be correlated to the maximum deflection magnitude (Figure 11b) instead of the total deflection magnitude. Similar to the loss-deflection relationships, high variations can be observed in the reflection intensity–deflection relationships of series 25D and 50D. Highly scattered results are observed for series 25D due to the strong effects of superposition of closely-spaced backscatter events and mode decoupling. Therefore, the reflection intensity may not be suitable for deflection measurement if closely spaced events are expected.

On the other hand, the mode dispersion increased with the fibre length and the mode decoupling triggered by the deflection as also observed in Figure 9. This can result in lower spatial resolutions of the OTDR signals and affect the detections and measurements of small deflection events.

## 4. Failure Monitoring

### 4.1. Specimens and Test Procedures

This section presents a study on different configurations of the POFs attached to concrete beams or failure monitoring. The goal is to maximise the sensitivity such that the crack/failure features can be readily identified. The normal strength (C30) concrete beams of 350 × 100 × 100 mm were pre-cracked of 2 mm width and 10 mm depth in the middle on the bottom face. Then four-point bending tests were performed on the beams with the loading applied on the top face and the bottom face supported near the edges.

**Figure 12:**
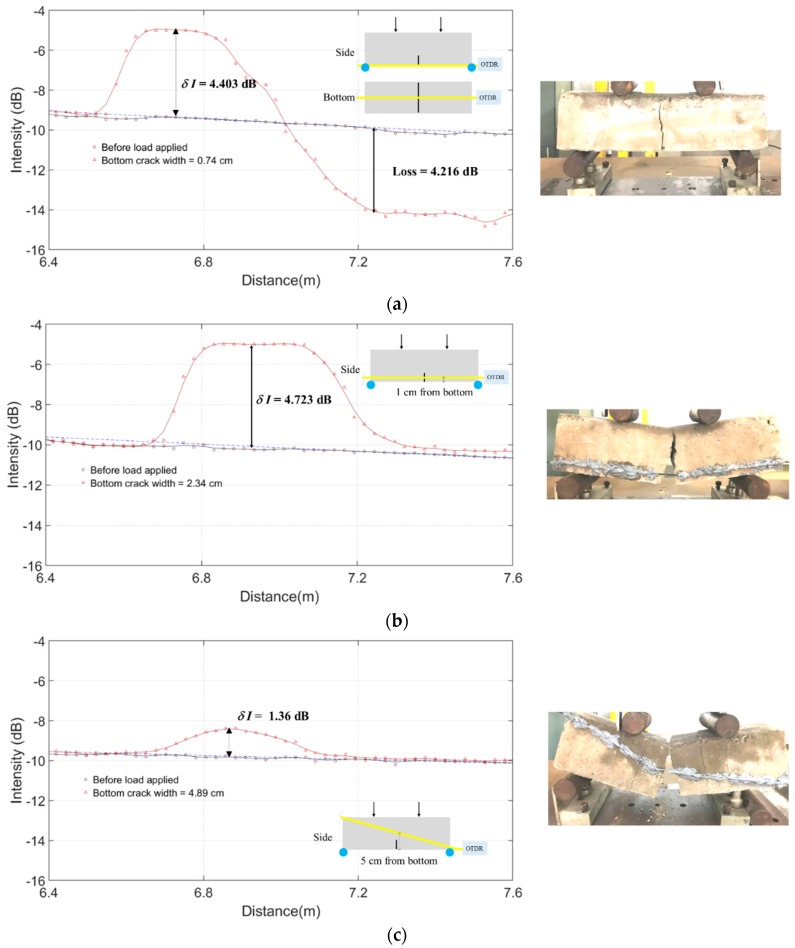

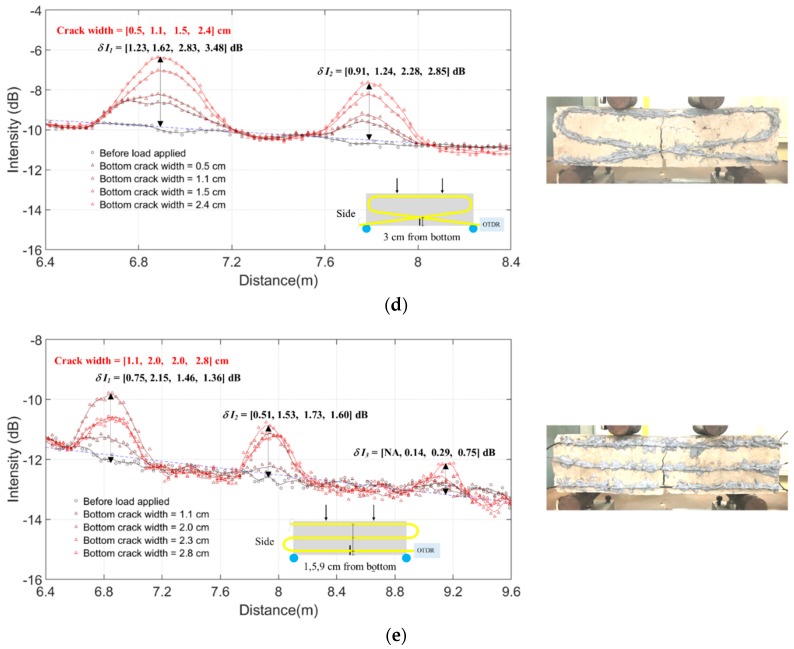
Monitoring of concrete beam failure in flexure: (**a**) a horizontal POF on the bottom face (specimen B); (**b**) a horizontal POF on the side face near the bottom edge (specimen SB); (**c**) a diagonal POF on the side face (specimen D); (**d**) two crossover POFs on the side face (specimen C2); (**e**) three parallel horizontal POFs on the side face (specimen S3).

Five configurations of POFs **(**Figure 12) attached on the beam surface with Sikadur-330 epoxy resin [39] were studied: (B) a horizontal POF on the bottom face along the centreline of the longitudinal direction; (SB) a horizontal POF on the side face which was 1 cm from the bottom edge (SB); (D) a corner to corner diagonal POF on the side face; (C2) two diagonal crossover POFs on the side face and the interception point was above the pre-crack of 3 cm from the bottom edge; (S3) three parallel horizontal POFs on the side face which were 1, 5, and 9 cm from the bottom edge, respectively. For specimens with a single POF (B, SB, and D), the sudden failure was observed, and hence, the traces were recorded only before the load applied and after the beam failure. The final bottom crack widths were recorded at the beam failure. For specimens (C2 and S3) with stable crack development, the traces were recorded for different bottom crack widths until the beam failure.

### 4.2. Test Results and Discussion

The flexural failure can be well detected and characterised in specimen B with the POF attached on the bottom face (Figure 12a). The highly localised curvature of the POF underneath the crack (crack width = 0.74 cm) at failure incurred significant bend losses. Meanwhile, despite the POF is attached close to the bottom of the beam in specimen SB (Figure 12b), the OTDR signals are quite different from that of specimen B. The POF was not bent but purely stretched along its axis that results in nearly no bend loss. Although the loss cannot be used to characterise the event, those unique features without significant loss but showing significant reflection can be used as an indicator for pure strain events.

In specimen D, as shown in Figure 12c, the POF was also purely stretched, and only the diagonal component of the crack opening was sensed by the POF. Therefore, the trace response was quite weak that even when the crack opening reached 4.89 cm, the reflection intensity (1.36 dB) was not even half of that for specimen SB (4.723 dB).

In specimen C2, the crack event occurred on the beam exactly at the interception of the two POFs (Figure 12d). The POFs were again not significantly bent but stretched as of specimen SB, so little loss was observed. This crossover POF layout toughened the beam, and hence, the crack developed gradually before the final failure. This allowed the traces to be captured for different values of crack widths in the same specimen.

In specimen S3 (Figure 12e), the three parallel POFs toughened the beam and the traces could also be captured for different cracking states. Contradicting to the observations in specimen C2, the reflection intensity of the first trace event (the crack event intercepted by the lowest POF) increase initially for crack width increasing up to 2 cm but decrease for further increase of the crack width to 2.3 cm and 2.8 cm. Such a counterintuitive phenomenon was due to the deboning of the POF from the epoxy resin, and hence, the actual strain in the POF reduced. It shows that the strain measurement would be significantly affected by the bonding strength between the POF and the structure. The second and third trace events were intercepted in the middle and the top POFs, respectively. Since the crack was propagating from the bottom to the top, they showed delayed responses to the bottom crack widths. Nevertheless, such layout could be useful for tracking the crack lengths or crack fronts besides quantifying the crack widths.

## 5. Conclusions

A new shear deformation measurement theory and algorithm based on the large beam deflection and optical bend loss theories were successfully developed for structural monitoring with polymer optical fibres (POFs) and the photon-counting ν-OTDR. The proposed technique was verified experimentally, and the following conclusions were drawn:

1. When the normalised shear deflections are larger than 0.2, the location and magnitude can be accurately estimated with the proposed technique. Nonetheless, for normalised shear deflections smaller than 0.2, the errors increased, but the event can still be detected and located with an absolute error of less than 0.5 m.
2. For closely spaced events with separation distance <0.25 m, the event areas would be overlapped, and only the leading event can be located. Nevertheless, the sum of all individual deflection magnitudes can still be evaluated from the total bend loss.
3. For well-separated events with separation distance >5 m, each shear event can be treated and analysed independently.
4. For failure monitoring, the OTDR signals increase with the localised bending of the POFs at the beam failure states.
5. The POFs attached on the sides of the beams that failed in flexure were purely stretched, and the corresponding OTDR traces had unique features with nearly no bend loss but high reflection intensity.
6. Due to the mode dispersion, the signal-to-noise ratio is lower for long-distance measurements, and that reduces the accuracy of detecting small deflection events.

## Figures and Tables

**Table 1:** Specifications of the ν-OTDRs [34] adopted in this study.

Specifications	Values/Types
Wavelength	650 nm or 520 nm
Optical pulse width	1 ns
Dynamic range	20 dB
Spatial resolution	Any multiple of 2.5 cm
Fibre type	Multimode PMMA 1 mm POF
Attenuation dead zones	40 cm (RL * = 45 dB); <1 m (RL = 14 dB)
Loss accuracy	± 0.1 ± 0.02 dB/dB
Measurement range	1.25 km

Notes: * RL = return loss = 10log10(reflected power/input power).

**Table 2:** Properties of the Polymethyl–Methacrylate Resin (PMMA) polymer optical fibres (POFs) adopted in this study.

	Core	Cladding	Jacket
Light propagation mode	Multimode with step refractive index profile		
Typical diameter (mm)	0.98	1	2.2
Material	Polymethyl–Methacrylate Resin (PMMA)	Fluorinated Polymer	High-Density Polyethylene
Typical refractive index	1.492	1.405	-
Typical Young’s modulus (GPa)	3.09	0.68	0.8
Maximum operation temperature	75 °C in a moist atmosphere and 85 °C in a dry atmosphere		
Typical weight (g/m)	0.88–0.91	0.09–0.12	3
Yield strength (MPa)	82		
Twisting endurance	5 times for loss increment ≤1 dB		
Repeated bending endurance	10,000 times for loss increment ≤1 dB		
Numerical aperture	0.5		
